# Intraocular Hemorrhage due to use of Sildenafil in a Patient with Diabetes

**Published:** 2018

**Authors:** Cagri Ilhan, Mehmet Murat Uzel, Mehmet Citirik

**Affiliations:** 1 Department of Ophthalmology, Hatay State Hospital, Hatay, Turkey; 2 Department of Ophthalmology, Afyonkarahisar State Hospital, Afyonkarahisar, Turkey; 3 Department of Ophthalmology, University of Health Sciences, Ankara Ulucanlar Eye Education and Research Hospital, Ankara, Turkey

**Keywords:** Sildenafil, Intraocular Hemorrhage, Type 2 Diabetes, Pars Plana Vitrectomy, Hyphema, Vitreous Hemorrhage

## Abstract

Sildenafil is one of the most commonly used drugs for sexual dysfunction or to increase libido, and it regulates endothelial nitric oxide synthase enzyme via selective phosphodiesterase-V inhibition. Sildenafil can be easily obtained without a medical indication or prescription yet it is not considered as a completely safe medication. Hemoptysis and hemorrhagic stroke are some important adverse effects of sildenafil. The case of the current report was a 67-year-old diabetic patient with simultaneous anterior and posterior segment hemorrhage after the use of 100 mg sildenafil citrate. Anterior chamber clearance and pars plana vitrectomy were performed for the patient because the hyphema and vitreous hemorrhage did not resolve during the follow-up period. There are very limited data available in the literature suggesting an increase in the risk of hyphema or vitreous hemorrhage due to the use of sildenafil. This is the first report that reveals the bleeding effect of sildenafil use in a patient with type 2 diabetes.

## INTRODUCTION

Sildenafil citrate is a highly selective phosphodiesterase-V inhibitor, which regulates the function of endothelial nitric oxide synthase enzyme [1]. It directly effects the vessels of the corpus cavernosum and is one of the most commonly used drugs for the treatment of sexual dysfunction and to increase libido and sexual performance [2, 3]. Sildenafil can be easily obtained with and without a prescription and is mostly used without medical supervision. Flushing, headache, and dizziness are the most prevalent side-effects of sildenafil and hemoptysis, and hemorrhagic stroke is one of the other important adverse effects [4-6]. The current study reports of simultaneous anterior and posterior segment hemorrhage due to the use of sildenafil in a patient with type 2 diabetes. 

## CASE REPORT

**Figure 1:**
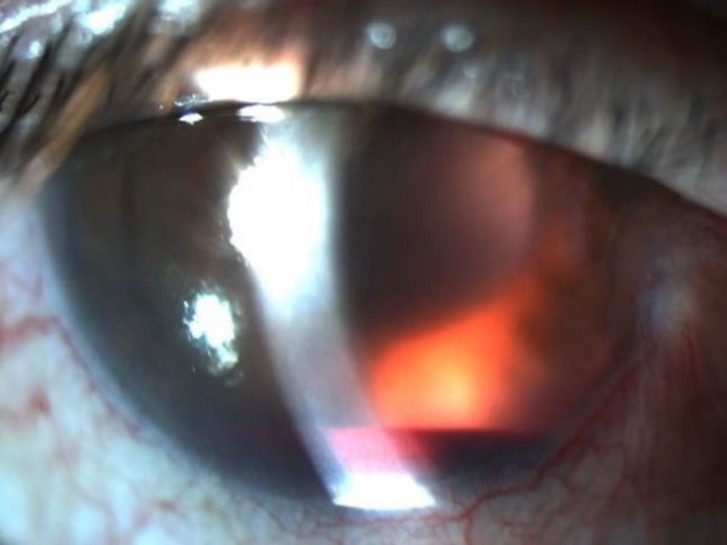
Anterior Segment Photograph of the Right Eye; Hyphema and the Red Reflex of the Intraocular Lens of the Vitreous Hemorrhage.

The case was a 67-year-old male patient with type 2 diabetes mellitus, who had complaints of sudden decreased vision in the right eye since the morning of referral. Corrected distance visual acuity in the right eye was hand motion and in the left eye, 20/40. Examination of the right eye revealed grade I hyphema, normal intraocular pressure, and normal appearance of iris tissue and posterior chamber intraocular lens, although the posterior segment was not illuminated. In ocular ultrasonography, the retina was normal yet the vitreous cavity was seen to be hyperechoic with high gain and preoccupied vitreous hemorrhage. In the left eye, there were hard exudates and dot-blot hemorrhages throughout the retina. In examinations of the left eye, optical coherence tomography revealed macular edema and the fluorescein angiogram revealed macular edema, microaneurysms, and focal leakage of vessels without any neovascularization. The patient stated that he used 100 mg of sildenafil citrate (Viagra; Pfizer, Istanbul, Turkey) the previous night for sexual stimulation and visual acuity of the right eye was good until this morning. The patient was asked about his medical history and drug use, and he reported that he had type 2 diabetes mellitus only and was only using insulin. The patient also stated that he had not received any other ocular treatment, such as retinal laser or intravitreal injection. The patient had worked regularly in the same job for the last five years and there were no important stressor factors in his life. An anterior segment photograph of the right eye was taken (Fig 1) and informed written consent was obtained for the academic use of the photograph.

**Figure 2:**
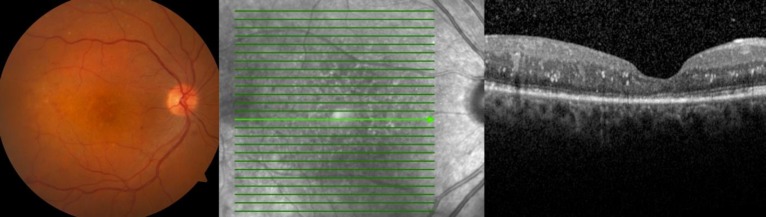
Intra-retinal Hemorrhages, Hard Exudates and Laser Scar can be shown in the First Postoperative Week Colored Fundus Photograph and Optical Coherence Tomography Image of the Right Eye

Therapy was firstly initiated with bed rest with the head in an upright position, 0.5% loteprednol etabonate ophthalmic suspension (Lotemax; Bausch & Lomb Pharmaceuticals, New Jersey, USA) four times in a day, and 1% tropicamide eye drop (Tropamid 1% Forte; Bilim İlaç Sanayi ve Ticaret AŞ, İstanbul, Turkey) two times a day. Anterior chamber clearance and pars plana vitrectomy were planned for the patient because the hyphema and vitreous hemorrhage did not resolve during the four weeks of follow-up. In the preoperative tests, the complete blood count and coagulation tests were determined to be completely normal and no disease other than type 2 diabetes mellitus was found in the internal medicine consultation. Pars plana vitrectomy was performed after one month and final corrected distance visual acuity increased to 20/40 during the first postoperative week. Intra-retinal hemorrhages, hard exudates, and laser photocoagulation scars were seen in colored fundus photograph and irregularities in retinal layers were shown in the optical coherence tomography image (Fig 2).

## DISCUSSION

In diabetic retinopathy, angiogenic mediators released from the ischemic retina cause fibro-vascular proliferation and the formation of neovascularization in the anterior (neovascularization of the iris) and posterior segments (neovascularization at the disc and neovascularization elsewhere) of the globe [7].

Fibrous component contraction can increase the tendency of these abnormal neovascular structures, leading to loss of vision due to bleeding in the anterior (hyphema) and posterior segments (vitreous hemorrhage) [8]. According to recent case reports, drug-induced intraocular hemorrhages are related to several drugs, such as aspirin, warfarin, dabigatran, and ginkgo biloba [9-12].

A number of articles have reported on blurred vision, color vision defects, retinal hemorrhage, and non-arteritic/arteritic ischemic optic neuropathy, as ocular side effects of sildenafil [13-16]. To the best of the author’s knowledge, this is the first report that mentioned intraocular hemorrhage due to the use of sildenafil in a patient with type 2 diabetes.

There have been numerous reports of intracerebral hemorrhage related to the use of sildenafil [17-19]. It is thought that the nitric oxide-cyclic guanosine monophosphate pathway, induced by inhibition of phosphodiesterase-V, leads to cerebral vasodilatation and increases blood flow. This mechanism increases the risk of intracranial hemorrhage [17]. The effects of sildenafil are not limited to vessels in the corpus cavernosum and central nervous system. Numerous symptoms, such as hemoptysis, purpuric dermatosis, dizziness, and stuffiness are related to the effects of sildenafil use on the lungs, skin, ear, and nose, respectively [4, 5, 20].

Sildenafil also has a vasodilator effect on the choroid and increases the volume of choroidal blood flow [21]. Koksal et al. [22] reported an increment in blood flow in both the ophthalmic and short posterior ciliary arteries after the use of sildenafil. When considering the combination of the effects of the presence of diabetic retinopathy and sildenafil use, the risk of intraocular hemorrhage is very high. 

Hyperglycemia causes loss of pericyte and thickening in basement membrane of retinal blood vessels while proliferative stage aberrant angiogenesis of retinal blood vessels results in vascular leakage, fibrovascular tissue formation, and vitreous hemorrhage [23]. The walls of abnormal neovascular structures are more sensitive to an increase of ocular blood flow, and bleed easily, triggered by the use of sildenafil. Generalized retinal ischemia and proliferative factors released from the retina, maintain the continuity of this pathophysiological course [24].

## CONCLUSION

The current case was a diabetic patient with unilateral hyphema and vitreous hemorrhage, who used sildenafil. There is no definitive evidence to confirm that the hemorrhage in this case was caused by sildenafil itself. Nevertheless, it can be hypothesized that the bleeding was related to sildenafil due to the former described mechanisms. To the best of the author’s knowledge, this is the first report of the development of hyphema and vitreous hemorrhage during the use of sildenafil in patients with type 2 diabetes. It can be speculated that the use of sildenafil may increase the risk of hyphema and/or vitreous hemorrhage in a patient with diabetes.
